# P55 The boggy uveitis

**DOI:** 10.1093/rap/rkae117.086

**Published:** 2024-11-05

**Authors:** Swapan Nagpal, Veer Singh

**Affiliations:** Sukh Sagar Hospital, Amritsar, India; S.B. Dr. Sohan Singh Eye Hospital, Amritsar, India

## Abstract

**Introduction:**

Blau syndrome is a rare autosomal dominant autoinflammatory disease that affects young children. Typically, it causes widespread granulomatous inflammation, causing uveitis, arthritis, and dermatitis. The triad may be characteristic of the disease but is not present in all cases and may occasionally be incomplete. A characteristic finding is the presence of boggy swellings in the involved joints. Our patient presented with **uveitis and boggy swellings of the wrists** without arthritis. The history of dermatitis was remote. A high clinical index of suspicion and exome sequencing are imperative to make this rare diagnosis.

**Case description:**

A six-year-old female was brought to an ophthalmologist with a complaint of a progressive decrease in vision in both eyes over the last four months. The vision deteriorated rapidly over the last couple of months. A detailed eye examination revealed features of granulomatous posterior uveitis with bilateral inflammatory cataracts. She was referred to the Rheumatology Outpatient Clinic. A detailed examination revealed boggy swellings on the bilateral wrist joints. As per the father, these swellings had been present for the last four years. The child never complained of pain in the wrists, or swelling and/or pain in any other joint. There was no history suggestive of arthritis or uveitis in the parents or immediate family members.

The child occasionally developed an itchy red rash on the back. Such a rash has not occurred for the last many months. There was no skin lesion at the time of the examination.

Suspecting Blau syndrome, a blood sample was sent for clinical exome sequencing (CES). Surgery (cataract extraction and intraocular lens implantation) was performed in the right eye. Intraoperative findings were noted, and features of granulomatous uveitis were evident. The patient was initiated on oral corticosteroids at 1 mg/kg of prednisolone by the eye surgeon. Four weeks later, the CES result was reported; it was positive for a heterozygous ‘pathogenic’ variant (detected in exon 4 of the NOD2 gene). The patient was reviewed after the report, and methotrexate was added. We started tapering the corticosteroids after six weeks of initial therapy. On the last eye examination (three months after the surgery), the uveitis had subsided. The vision was normal in the operated eye and had considerably improved in the unoperated eye. The surgery for the left eye was now deemed non-obligatory and was thus deferred.

**Discussion:**

Blau syndrome is a rare autosomal dominant syndrome that causes arthritis, dermatitis, and granulomatous uveitis in children. A characteristic clinical feature of Blau syndrome is the presence of boggy swellings in the involved joints. Skin involvement usually occurs before the onset of arthritis and uveitis, presenting in the form of a rash (macro or micropapular and scaly), and occurs in infancy. Arthritis appears subsequently (between two and four years of age), followed by uveitis (around four years of age). Other uncommon manifestations of this disease are lymphadenopathy, sialadenopathy, erythema nodosum, neuropathies, leukocytoclastic vasculitis, glomerular and interstitial nephritis, interstitial lung disease, and pericarditis. A close mimic of this disease is sarcoidosis. Blau syndrome is an autosomal dominant disorder due to a mutation in the caspase recruitment domain gene CARD15/NOD2.

There is no consensus on the optimal treatment for Blau syndrome. Corticosteroids and immunosuppressive agents, including methotrexate and azathioprine, have been used. Biologic agents have also been used with good results.

In our case, the patient never had joint pains. He did not seek help until the child’s vision was impaired. Commonly, the ophthalmologist may be the only qualified physician of first contact. Joint pains may often be treated with self-medication or may be labelled as “growing pains” but the loss of vision is an unignorable catastrophic symptom and is invariably reported. The alert eye specialist must also enquire about fever, arthritis, and dermatitis in children with granulomatous uveitis. This correlation must be made sooner rather than later to prevent delay in diagnosis and therapy.

The temporal dispersion of symptoms, i.e., the occurrence of different symptoms at different ages in the affected individual, also makes the diagnosis of Blau syndrome very challenging.

**Key learning points:**

• Blau syndrome is a rare autosomal dominant disorder due to a mutation in the caspase recruitment domain gene CARD15/NOD2.

• It causes arthritis, dermatitis, and granulomatous uveitis.

• A characteristic clinical feature of Blau syndrome is the presence of boggy swellings in the involved joints.

• A strong index of suspicion is required for diagnosis of this rare disorder.

